# Developing methods to identify resilience and improve communication about diagnosis in pediatric primary care

**DOI:** 10.3389/fmed.2024.1414892

**Published:** 2024-09-30

**Authors:** Irit R. Rasooly, Trisha L. Marshall, Christina L. Cifra, Ken Catchpole, Nicholas C. Kuzma, Patrick W. Brady, Katherine Melton, Alisa Khan, Alyna T. Chien, Ellen A. Lipstein, Christopher P. Landrigan, Kathleen E. Walsh

**Affiliations:** ^1^Clinical Futures: A Center of Emphasis within the CHOP Research Institute, Children’s Hospital of Philadelphia, Philadelphia, PA, United States; ^2^Department of Pediatrics, Perelman School of Medicine at the University of Pennsylvania, Philadelphia, PA, United States; ^3^Division of Hospital Medicine, Cincinnati Children’s Hospital Medical Center, Cincinnati, OH, United States; ^4^Department of Pediatrics, College of Medicine, University of Cincinnati, Cincinnati, OH, United States; ^5^Division of Medical Critical Care, Department of Pediatrics, Boston Children’s Hospital, Boston, MA, United States; ^6^Department of Pediatrics, Harvard Medical School, Boston, MA, United States; ^7^Department of Anesthesia and Perioperative Medicine, Medical University of South Carolina, Charleston, SC, United States; ^8^Drexel University College of Medicine, Philadelphia, PA, United States; ^9^Department of Pediatrics, St. Christopher’s Hospital for Children, Philadelphia, PA, United States; ^10^James M. Anderson Center for Health Systems Excellence, Cincinnati Children’s Hospital Medical Center, Cincinnati, OH, United States; ^11^Division of General Pediatrics, Department of Pediatrics, Boston Children's Hospital, Boston, MA, United States; ^12^Division of Sleep and Circadian Disorders, Departments of Medicine and Neurology, Brigham and Women’s Hospital, Boston, MA, United States; ^13^Department of Pediatrics, Department of Medicine, and Division of Sleep Medicine, Harvard Medical School, Boston, MA, United States

**Keywords:** diagnosis, diagnostic process, patient safety, safety II, pediatrics, communication, primary care, ambulatory care

## Abstract

Communication underlies every stage of the diagnostic process. The Dialog Study aims to characterize the pediatric diagnostic journey, focusing on communication as a source of resilience, in order to ultimately develop and test the efficacy of a structured patient-centered communication intervention in improving outpatient diagnostic safety. In this manuscript, we will describe protocols, data collection instruments, methods, analytic approaches, and theoretical frameworks to be used in to characterize the patient journey in the Dialog Study. Our approach to characterization of the patient journey will attend to patient and structural factors, like race and racism, and language and language access, before developing interventions. Our mixed-methods approach is informed by the Systems Engineering Initiative for Patient Safety (SEIPS) 3.0 framework (which describes the sociotechnical system underpinning diagnoses within the broader context of multiple interactions with different care settings over time) and the Safety II framework (which seeks to understand successful and unsuccessful adaptations to ongoing changes in demand and capacity within the healthcare system). We will assess the validity of different methods to detect diagnostic errors along the diagnostic journey. In doing so, we will emphasize the importance of viewing the diagnostic process as the product of communications situated in systems-of-work that are constantly adapting to everyday challenges.

## 1 Introduction

Communication underlies every stage of the diagnostic process. As patients and families are present throughout the diagnostic process, effective communication with patients and families is both a definitional aspect of diagnostic excellence (1) and a pragmatic strategy for achieving it. Due to limitations of the current healthcare system, including fragmented outpatient care and limitations to access for patients who speak languages other than English, patients and families are often the ones communicating clinical information from one diagnostic encounter to another (2, 3). Research engaging families in safety reporting identified that families uncovered multiple diagnosis-related errors and adverse events, including delayed diagnoses of intussusception, aspiration pneumonia, and urosepsis (4, 5). The quality of bidirectional communication between patients, families/caregivers, and clinicians determines how diagnostic information is gathered, integrated into differential diagnoses, and communicated back to patients/caregivers, ultimately contributing to diagnostic outcomes. Yet, in spite of their important role in diagnostic communication, studies characterizing opportunities to improve diagnosis often lack the patient and family view (4, 6, 7).

In recognition of the critical shared role of patients, their families, and clinicians in diagnosis, the National Academy of Science, the National Quality Forum, the Institute for Healthcare Improvement, the US Center for Medicare & Medicaid Services, and the Children’s Hospital Association have all called for improved patient engagement in the diagnostic process (1, 6, 8, 9). Engaging patients more closely in diagnostic and safety research is respectful, ethical, and ensures safer care (10–12). Robust communication between patients, families/caregivers, and clinicians is more likely to generate resilience and safeguard against diagnostic failures and harm. Conversely, communication failures contribute to nearly half of malpractice claims, with more than half being provider-patient communication failures (13). In particular, there is ample opportunity to improve effective communication about diagnostic uncertainty (14–17).

Vulnerable populations, including families from racial/ethnic minority groups, those with lower incomes, and those who speak languages other than English disproportionately experience communication failures in health care (7, 18). Disparities have been characterized in the diagnosis of varying conditions including depression, appendicitis, acute myocardial infarction, and breast cancer (19–23). It is particularly important to understand how pediatric diagnostic processes differ for families who speak languages other than English. For example, patients with limited English proficiency are four times as likely to report having no qualified provider or interpreter who spoke their language, three times as likely to report incorrect or out of date medical records, and twice as likely to report not understanding the follow-up plan (24).

Effective communication is imperative and challenging in pediatrics, where different parents and/or other caregivers often hand off responsibility for clinic appointments or childcare. Often, children have a limited ability to convey symptoms, relying on multiple family caregivers. This is particularly true among children with complex or disabling conditions who average 6.5 (SD 6.5) outpatient visits annually, most to primary care (25). Most of these children (60%) will see a specialist at least once a year, 20% will visit the emergency room, and 10% will be admitted to the hospital (25, 26).

Diagnostic errors have been defined by the National Academy of Medicine as, “the failure to (a) establish an accurate and timely explanation of the patient’s health problem(s) or (b) communicate that explanation to the patients. This definition underscores this critical component that communication plays in avoiding delayed, missed, or wrong diagnoses. Opportunities to improve pediatric diagnosis, in terms of accuracy or communication, have not been well characterized (8). While an estimated 5% of adult primary care visits involve a diagnostic error (27), rates and characteristics of diagnostic errors are largely unquantified and undescribed in pediatric primary care. Research in specific disease conditions suggest that opportunities to improve pediatric diagnosis are common. Studies of hypertension or adolescent depression found that these diagnoses are missed 50% of the time in pediatric primary care (28). Up to 20% of children also have a delayed diagnosis of physical abuse (29–33). Diagnostic errors also occur with other common pediatric conditions, with misdiagnosis reported in 14% of asthma and 8% of appendicitis diagnoses (8). In a national study of pediatric health system leaders and parents, diagnostic safety was identified as a high priority topic for research (34). There is an urgent need to evaluate and develop interventions to improve diagnostic communication to prevent ongoing injury and death from diagnostic errors. This need is particularly urgent in primary care, where most of pediatric healthcare is delivered.

In this manuscript, we will describe the theoretical frameworks, protocols, data collection instruments, methods, and analytic approaches to be used in the Dialog Study. This study aims to characterize the pediatric diagnostic journey, focusing on communication as a source of resilience, in order to develop and adapt structured, patient-centered communication interventions for outpatient use, and to test the efficacy of such interventions in improving diagnostic safety. We will attend to patient and structural factors, like race and racism, and language and language access, before developing interventions.

## 2 Theoretical frameworks

Our mixed methods approach is grounded in the Systems Engineering Initiative for Patient Safety (SEIPS) 3.0 framework (35) which describes the sociotechnical system within the broader context of multiple clinical interactions that patients have in different care settings over time. We aimed to understand both the successful and unsuccessful adaptations to ongoing changes in demand and capacity within the healthcare system (36).

Recognizing that healthcare is “increasingly distributed over space and time,” Carayon et al. recently updated her widely used Systems Engineering Initiative for Patient Safety (SEIPS) framework, to characterize the patient journey in multiple care settings over time (35). Generally, the SEIPS framework allows for analysis of patient outcomes as a product of the interaction between structures (people, environment, tasks, tools) and processes (37, 38). Grounding the understanding of diagnostic safety as a longitudinal journey constructed by the dynamic interplay of sociotechnical elements will guide our observations, simulations, and analysis.

Our study investigates the diagnostic journey through both a traditional “error” perspective (Safety I) and approaches to avert errors and achieve diagnostic excellence (Safety II). Identifying both opportunities to improve diagnosis and drivers of diagnostic excellence is aligned with principles of “resilience engineering,” which posit that safety is a consequence of adapting to the changing conditions of systems function (39). In other words, harm occurs not because an otherwise stable system malfunctioned but rather because inappropriate adaptive actions were taken within an ever-changing, inherently error-prone environment. Thus, resilient systems can (1) respond (*know what to do*), (2) monitor (*know what to look for*), (3) learn from experience, and (4) anticipate (*know what to expect*) (40). We will assess the presence and interplay of these factors and the role of robust communication in their presence along the diagnostic journey.

SEIPS 3.0 and Safety II align to overcome limitations of approaches that seek improvement by rectifying piecemeal individual errors to instead explore how resilient people and systems deliver appropriate diagnoses and what systems barriers and facilitators shape these processes. It allows for the prospective identification of the everyday practices that contribute to effective diagnosis, rather than relying on retrospective assessment of failures. Informed by these frameworks, we will assess the validity of different methods to detect diagnostic errors along the diagnostic journey. In doing so, we will extend the idea that the diagnostic process is the product of communications situated in systems-of-work that are constantly adapting to everyday challenges.

## 3 Methods and analysis

### 3.1 Study design

This is a prospective, mixed-methods, observational study aimed at characterizing opportunities to improve diagnosis and sources of systems resilience that drive diagnostic excellence for children with medical complexity presenting to primary care with acute concerns. We will adapt and test well-established patient safety research methods (including chart review, hospital incident reporting, family safety reporting, surveys, observations, and interviews) to characterize the diagnostic journey and associated successes and harms experienced by acutely ill children with multiple comorbid conditions and their families (Table 1). We will also apply reliable, valid, and novel ethnographic methods developed initially to understand the high rates of errors at home among outpatient children with chronic conditions (Table 1) (41). Ultimately (in subsequent phases of this initiative) findings will inform development and piloting of a communication-based intervention to improve diagnostic safety in this population.

**Table 1:** Study procedures, timing and purpose of each procedure.

Procedure	Timing	Purpose
**Observation of urgent visit** (*n* = up to 35)*	Presentation for urgent problem	Evaluate patient-centered communication quality and content and diagnostic uncertainty. Identify interventional opportunities.
**Parent interview** (*n* = up to 35)*	Within 2 weeks of observation	Assess patient-centered communication quality and content, diagnostic uncertainty, and discrimination
**Clinician interview** (*n* = up to 35)*	Within 2 weeks of observation	Evaluate patient-centered communication quality and content and diagnostic uncertainty
**Chart review** (*n* = 150)	Within 4 weeks, and by 6 months after the last visit for presenting problem	Describe the diagnostic journey. Identify diagnostic success and errors applying standardized patient safety and diagnostic evaluation instruments.
**Parent phone survey** (*n* = 150)	Within 4 weeks, and by 6 months after the last visit for presenting problem	Identify elements of the diagnostic journey not recorded in the chart. Identify diagnostic success and error.

*Observations and interviews will be performed until thematic saturation is achieved.

A diagnostic journey as conceptualized in SEIPS 3.0 and the National Academies of Medicine starts at the first symptoms of a new problem at home leading to engagement with the healthcare system (1, 35). The healthcare team then engages in an iterative process of information gathering, information integration/interpretation, and formulation of a working diagnosis which is communicated to the patient and family and revisited as needed in response to treatment; success in this process determines outcomes for both the patient and system (1).

We will sample children followed at our primary care or complex care clinics (designed to care for patients with multi-specialty involvement, technology dependence, and/or neurologic impairment). *Our preliminary chart review showed that diagnostic journeys for children with multiple chronic conditions lasted from one day to three weeks.* We thus expect to capture the majority of diagnostic errors within six weeks of the initial presenting visit and will perform chart reviews and parent/caregiver phone surveys at that time. We will re-review all charts six months later to characterize the distribution of the duration of the diagnostic journey and assess the sensitivity of the six-week cutoff.

### 3.2 Setting

Data will be collected in five primary care and complex care ambulatory clinics associated with three children’s hospitals in the Northeast and Midwest. Clinic patient characteristics vary. Between 40 and 6% of patients identify as Hispanic, 45 to 27% of patients identify as Black, and 69 to 23% of patients identify as white. The majority of patients have public insurance (range across clinics 55 to 85%). Parental preference for a language other than English ranges from 20 to 8%.

### 3.3 Participant selection

We will recruit 150 patients age < 21 years with multiple chronic conditions (excluding mild asthma, eczema, allergies) presenting with irritability, vomiting, fever, abdominal pain, or other acute illness. We will review records of and conduct a brief phone survey (family safety reporting) with all participants. For the qualitative phase of the study, we will observe up to 35 of these patients’ clinic visits and invite parents/caregivers and a clinician from each observation to participate in interviews and additional, follow up phone surveys. To assure a comprehensive perspective on the diagnostic process, we will only include patients who receive primary care within our health systems. To understand differences in diagnostic processes by patient/family characteristics and parent/caregiver language, we will recruit patients with diverse racial and ethnic backgrounds and oversample patients with caregivers who prefer Spanish. Research assistants from diverse racial, ethnic, and language backgrounds will recruit outpatients by phone or in person. Participants will be compensated for interviews and observations. Consent will be obtained; assent will be obtained from the child where appropriate.

### 3.4 Methods

**Chart reviews and brief phone surveys** (150 patients) will be conducted to understand the longitudinal diagnostic journey, identify medical errors/harms, and characterize opportunities to improve diagnosis. Initial reviews will take place within 4 weeks of an acute care visit and will continue, with the goal of characterizing the diagnostic journey in its entirety, for up to 6 months. To further characterize the diagnostic journey, a trained research nurse will abstract the dates and locations of interactions with the health system, including in person, virtual, telephone, and portal message encounters. All pertinent communication and documentation regarding presenting, index-visit symptoms will be abstracted. We will detail “good catches” or “near misses” as sources of resilience. Charts will be reviewed for medical errors, associated harms, and harm severity (2, 42–47). In order to evaluate diagnosis, a structured EHR review tool [Revised SaferDx (48, 49)] will be applied to each case by two clinicians who will determine whether there were missed opportunities to improve diagnosis. Missed opportunities will be further classified using the modified DEER taxonomy to identify the phase of the diagnostic process involved (50). To maximize reliability, reviewers will receive didactic training practice on identical charts (45, 46). We will assess inter-reviewer reliability and discordant reviews will be adjudicated by a third, consensus reviewer.

In light of the inherent limitations of retrospective chart review, we will also conduct brief phone surveys with patients/families to identify aspects of the diagnostic journey not otherwise captured in the medical record including parent/caregiver perceptions of diagnostic excellence, error, communication, and systems resilience. Parent/caregiver phone surveys are routinely used in outpatient safety research to identify care processes and potential errors not recorded in the chart (51, 52). We developed a ten-minute, 15-item phone survey, adapting from previously developed tools to capture family safety reporting in the hospital (4, 5, 53, 54) The survey examines points along the diagnostic journey, including successes, failures, and errors at each point. Surveys will occur within 4 weeks, and by 6 months after the last known point of contact with the health care system for the diagnostic journey (e.g., last outpatient visit, last outpatient follow up after hospitalization, or hospital discharge).

**Figure 1:**
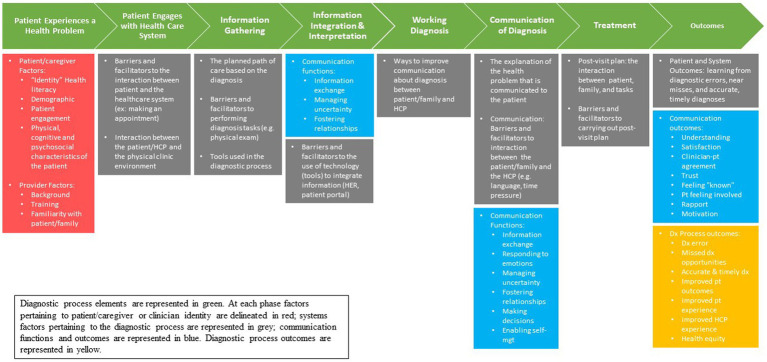
Integrated conceptual model of the ways patient/caregiver identity, clinician-patient communication health outcomes, resilience engineering, and the systems engineering for patient safety framework intersect and should be evaluated during the diagnostic process.

**Observations**. A subset of up to 35 patients will be recruited to participate in ethnographic observations. Observations will aim to characterize variation in diagnosis-focused communication across the diagnostic process (1) including during the phases of information gathering, information interpretation and integration, and formulation/communication of the working diagnosis (and diagnostic uncertainty, if present). A trained research assistant will be physically present in the exam room, directly observe, and audio-record the patient’s entire clinic visit. The Hawthorne effect will be mitigated by having ethnographers shadow providers right before recruitment starts to increase clinic staff’s familiarity with the research team, training ethnographers to be inobtrusive, and triangulating data from observations with other data collected from interviews and chart review. We will utilize an iteratively-developed observational guide developed based on conceptual models of patient/family identity, clinician-patient communication and health outcomes (55), Safety II/resilience engineering (56), and the SEIPS 3.0 framework (35) (Figure 1). Portions of audio-recorded observations will be transcribed at a later date, if needed to understand communication content. Diagnostic successes and errors will be identified within and across SEIPS work system domains (37) and used to model the system and identify resilience (36).

**Semi-structured interviews** of parents/caregivers and clinicians who participate in observations will be performed by a trained interviewer in person or by phone, within 2 weeks of observation. The goal of these interviews is to reflect on interactions and processes during the observed clinic visit in order to obtain a deep understanding of how communication affects the diagnostic processes in light of patient and family characteristics, circumstances, and values. Interviews will explore parent/caregiver experiences in accessing care, perceived discrimination, and patient-centered communication (“*When did the doctors listen to you?” “not listen*?”) throughout the diagnostic process, from history taking to formulation of working diagnosis. Interviews may also ask about adaptive clinician approaches, and variation in parent/caregiver “speaking up.” The semi-structured interview guides were developed using the same frameworks as for the observation guide and will be pilot-tested with diverse participants and edited as needed for clarity. Interviews will be conducted by trained bilingual interviewers, audio-recorded, and transcribed by a HIPAA-compliant transcription and translation service. Surveys, administered concurrent to the interviews, will inform interpretation of qualitative data. Parents/caregivers will compete a 3-item health literacy survey (57). Clinicians participating in ethnography will complete the Team Dynamics survey (58).

### 3.5 Analysis of chart reviews

Descriptive statistics will be used to summarize demographics and aspects of diagnostic journeys, including duration (in days), number and types of contacts within healthcare, number of people, and clinics involved. Rates of medical and diagnostic errors will be estimated. Patient-days will be determined by calculating the number of days between first point of contact with the health system and the most-definitive diagnosis and treatment. Rates will be calculated per patient and per 1,000 patient-days. Tabulation of SaferDx (49) and DEER taxonomy (50) results will be used to ascertain rates and types of diagnostic successes and errors. In addition to establishing a baseline incidence of opportunities to improve diagnosis in this primary care cohort, our analysis will identify areas of vulnerability in the diagnostic process and elucidate the relationship between medical errors, diagnostic errors, and outpatient harm. In conjunction with qualitative analysis, our findings will focus and direct interventional efforts targeting opportunities identified in the evaluation of existing diagnostic processes.

Because of the small sample size, we will perform stratified analyses by site only and will not create adjusted models. Given the size of this study and the expected rate of diagnostic errors, we do not expect to quantitatively identify differences in rates of diagnostic errors by parent/caregiver preferred language, insurance status, or self-reported cultural, religious, gender, disabilities, or racial/ethnic identities. However, we do expect to note qualitative differences in diagnostic processes, which will inform future observational and interventional studies. Demographics of participants will be compared with those of eligible non-participants who declined or whom we were unable to consent for participation in order to evaluate for potential selection bias.

### 3.6 Qualitative analysis of observations and semi-structured interviews

Qualitative analysis will occur in tandem with ongoing observations and interviews to ensure fidelity to the guides, appropriate observation and interviewing techniques, and to enable detection of data saturation (the point at which no new major codes/themes emerge). Analyses will proceed through the development of mixed deductive/inductive codes and coding using the constant comparative method, culminating in overall thematic analysis of the coded data (59). We will develop deductive codes drawing on the literature and conceptual models of patient/family identity clinician-patient communication (55), Safety II (56), and the SEIPS 3.0 framework (35) used to generate the observation guide (Supplemental). Qualitative coding will be performed using Dedoose software (Version 9.0.17, SocioCultural Research Consultants, LLC, Los Angeles, CA, 2021) by assigning deductive (from *a priori* codebook) and inductive codes (from emergent themes) to text segments, grouping related codes, and iteratively revising the coding structure as new codes emerge. Group coding will be performed for qualitative data from the first 2–3 patients, revising the codebook as needed. Thereafter, qualitative data from each patient will be coded independently by two coders, resolving discrepancies at scheduled consensus meetings. We will develop definitions for diagnostic success and errors and use inductive thematic analysis to ascertain systems factors that appear to be contributing to the events. Directed content analysis will be used to identify the ways in which patients, families, and clinicians communicate along the diagnostic journey within and between systems of care. We will note successes, sources of resiliency, errors, and barriers or facilitators of the diagnostic process. We will compare processes and themes along major sub-groups. We will also characterize the diagnostic system, processes, and resilience at each site, comparing the similarities and differences, and thus identifying common themes and specific features.

Our ethnographic and chart review data will inform the development of process maps showing the entire diagnostic journey for children with multiple chronic conditions at each site and across sites, as well as process maps for families using Spanish for care. In our prior studies, such process maps have been pivotal in identifying points for intervention. Moreover, analysis of communication, patient/family-reported opportunities to improve diagnosis, and identification of the sources of resilience that can facilitate diagnostic excellence will focus intervention design on key aspects of diagnostic process.

## 4 Ethics and dissemination

Findings resulting from the described protocol will provide a basis for the development of interventions and methods to be used broadly in evaluating and improving the provision of diagnostic excellence in pediatric primary care. This study poses minimal risk to participants and has been approved by the single Institutional Review Board at Boston Children’s Hospital upon which the other sites are reliant. Consent and assent will be obtained as described above. In the unlikely event that we identify a serious diagnostic error in evolution or adverse event in evolution, we will address these directly with the clinical team, including the attending physician. The clinical team would follow their usual clinical procedure, including following institution-specific guidelines for error reporting.

## 5 Discussion

Our methods and outcomes are novel and represent an effort to evaluate and improve diagnostic communication longitudinally across time and care settings. Findings will help hone our understanding of the relationships between pediatric patients with medical complexity and clinicians, the ways patient/caregiver racial, ethnic, and linguistic identities intersect with communication and diagnostic outcomes, and the aspects of care that enable diagnostic excellence. With ethnography we will be able to evaluate systems resilience and when diagnostic communication processes “go well,” using a Safety II lens. In doing so, we will extend the idea that the diagnostic process is the product of communications situated in systems-of-work that are constantly adapting to the everyday challenges. Both ethnographic and chart review methods are meant to test and refine tools for identifying opportunities to improve diagnosis (Safety I). More broadly, we will assess the validity of different methods to detect diagnostic errors along the diagnostic journey. An outcome of this work will be evaluation and reflection on our methodology for detecting systems resilience, for detecting opportunities to improve diagnosis in primary care, for achieving across-site concordance in identifying opportunities to improve diagnosis, and for leveraging interdisciplinary expertise to evaluate cases. Our approach will allow us to learn about the diagnostic process for these vulnerable patients in primary care, but also extend and refine methods to be used to evaluate diagnostic performance and equity.
